# F123. BELIEFS ABOUT THEIR VOICES AND DEGREE OF RESILIENCE IN PERSONS WITH AUDITORY VERBAL HALLUCINATIONS WITH AND WITHOUT NEED FOR CARE

**DOI:** 10.1093/schbul/sby017.654

**Published:** 2018-04-01

**Authors:** Julien Laloyaux, Alberto Collazzoni, Isabella Kusztrits, Marco Hirnstein, Kristiina Kompus, Josef J Bless, Frank Larøi

**Affiliations:** 1 University of Bergen, NORMENT – Norwegian Center of Excellence for Mental Disorders Research, University of Oslo, University of Liège; 2 University of California, Los Angeles; 3 University of Bergen; 4 University of Bergen; NORMENT – Norwegian Center of Excellence for Mental Disorders Research, University of Oslo

## Abstract

**Background:**

Auditory Verbal Hallucinations (AVH) are prevalent in many psychopathologies but are also experienced by a minority of the healthy general population. There is cumulative evidence that the beliefs people hold about their voices (e.g., power) are strongly related to the impact of the voices (e.g., depression, anxiety) and to the coping strategies that they adopt (e.g., resistance, engagement). To date, research on resilience has identified many factors that promote wellbeing and that protect people from developing psychopathologies despite exposure to health or psychological adversities. However, no previous studies have examined resilience in people who experience AVH with and without need for care, and neither have the relations between resilience and beliefs about the voices been examined.

**Methods:**

Fifty persons who report hearing voices frequently were recruited online. Based on the presence of a psychiatric diagnosis, the use of antipsychotic medication, and on the consultation of a psychiatrist, they were then classified as being Healthy Voice-Hearers (HVH) or Patients (P). One hundred and nineteen healthy participants who have never experienced hearing voices were also recruited as a control group (CTRL). All participants completed the Resilience Scale for Adults. In addition, the HVH and P groups completed questionnaires that assess the beliefs they hold about their voices (the revised Beliefs About Voices Questionnaire) and voice characteristics (frequency and emotional content).

**Results:**

The data collection is currently underway, and thus the following results are preliminary. Kruskal-Wallis ANOVAs revealed significant differences between the three groups (HVH, P, CTRL) on several resilience factors. In particular, post-hoc analyses demonstrated that the CTRL and HVH groups were more resilient than the P group for the perception of self and of future. In addition, the HVH group was found to be more resilient than the P group in terms of social competence. Finally, for social factors (social resources and family cohesion), results showed that the CTRL group was more resilient than the P group. However, the HVH group was not significantly different from the P and the CTRL groups. Concerning voice characteristics, Mann-Whitney tests revealed that, compared to the P, the HVH perceived their voices as being less omnipotent and malevolent, less negative and more positive, and showed less resistance against the voices. Finally, correlational analyses (Spearman) demonstrated that better resilience (and in particular the individual factors such as the perception of self and of the future, and social competence) was related to fewer negative beliefs about the voices, less resistance, lower voice frequency, and less negative and more positive emotional content.

**Discussion:**

The present study showed that people who experience AVH without need for care have a different pattern of resilience compared to patients with AVH, and to healthy controls without AVH. In particular, the HVHs did not differ from the CTRL on the personal factors of resilience and did not differ from the patients in terms of social factors. In addition, better resilience (and especially the personal factors) was found to be related to fewer negative beliefs about the voices, better coping strategies, lower voice frequency, and less negative and more positive emotional content. Taken together, these results show that resilience – and in particular, the personal factors – may be an important variable influencing the need for care in people experiencing AVH. The present study has important theoretical and clinical implications, in particular, suggesting that the personal factors of resilience may be a treatment target in order to diminish the impact of voices.

